# *Pseudomonas aeruginosa* Susceptibility in Spain: Antimicrobial Activity and Resistance Suppression Evaluation by PK/PD Analysis

**DOI:** 10.3390/pharmaceutics13111899

**Published:** 2021-11-08

**Authors:** Ana Valero, Alicia Rodríguez-Gascón, Arantxa Isla, Helena Barrasa, Ester del Barrio-Tofiño, Antonio Oliver, Andrés Canut, María Ángeles Solinís

**Affiliations:** 1 Pharmacokinetic, Nanotechnology and Gene Therapy Group (PharmaNanoGene), Faculty of Pharmacy, Centro de Investigación Lascaray Ikergunea, University of the Basque Country UPV/EHU, Paseo de la Universidad 7, 01006 Vitoria-Gasteiz, Spain; avalero@fhp.cat (A.V.); alicia.rodriguez@ehu.eus (A.R.-G.); arantxa.isla@ehu.eus (A.I.); 2 Pharmacy Service, Fundació Sant Hospital, Passeig Joan Brudieu 8, 25700 La Seu d’Urgell, Spain; 3 Bioaraba, Microbiology, Infectious Disease, Antimicrobial Agents, and Gene Therapy, 01006 Vitoria-Gasteiz, Spain; 4 Instituto de Investigación Sanitaria Bioaraba, 01006 Vitoria-Gasteiz, Spain; helena.barrasagonzalez@osakidetza.eus; 5 Intensive Care Unit, Araba University Hospital, Osakidetza Basque Health Service, 01006 Vitoria-Gasteiz, Spain; 6 Microbiology Service, Hospital Son Espases, Instituto de Investigación Sanitaria Illes Balears (IdISBa), 07120 Palma de Mallorca, Spain; ester.delbarrio@ssib.es (E.d.B.-T.); antonio.oliver@ssib.es (A.O.); 7 Microbiology Service, University Hospital of Araba, Osakidetza Basque Health Service, 01006 Vitoria-Gasteiz, Spain

**Keywords:** *Pseudomonas aeruginosa*, pharmacokinetic/pharmacodynamic (PK/PD) analysis, Monte Carlo simulation, antimicrobial resistance, probability of target attainment (PTA), cumulative fraction of response (CFR)

## Abstract

*Pseudomonas aeruginosa* remains one of the major causes of healthcare-associated infection in Europe; in 2019, 12.5% of invasive isolates of *P. aeruginosa* in Spain presented combined resistance to ≥3 antimicrobial groups. The Spanish nationwide survey on *P. aeruginosa* antimicrobial resistance mechanisms and molecular epidemiology was published in 2019. Based on the information from this survey, the objective of this work was to analyze the overall antimicrobial activity of the antipseudomonal antibiotics considering pharmacokinetic/pharmacodynamic (PK/PD) analysis. The role of PK/PD to prevent or minimize resistance emergence was also evaluated. A 10,000-subject Monte Carlo simulation was executed to calculate the probability of target attainment (PTA) and the cumulative fraction of response (CFR) considering the minimum inhibitory concentration (MIC) distribution of bacteria isolated in ICU or medical wards, and distinguishing between sample types (respiratory and non-respiratory). Ceftazidime/avibactam followed by ceftolozane/tazobactam and colistin, categorized as the Reserve by the Access, Watch, Reserve (AWaRe) classification of the World Health Organization, were the most active antimicrobials, with differences depending on the admission service, sample type, and dose regimen. Discrepancies between EUCAST-susceptibility breakpoints for *P. aeruginosa* and those estimated by PK/PD analysis were detected. Only standard doses of ceftazidime/avibactam and ceftolozane/tazobactam provided drug concentrations associated with resistance suppression.

## 1. Introduction

The World Health Organization (WHO) has declared antimicrobial resistance as one of the top 10 global public health threats facing humanity [1]. The emergence of resistance to multiple antimicrobial agents in pathogenic bacteria has become a significant public health concern, as there are few, or even sometimes a complete lack of, effective antimicrobial agents available for infections caused by these bacteria. This fact is especially relevant considering that, in the last 10 years, no new group of antibiotics has been marketed in Europe [2].

*P. aeruginosa* is an opportunistic pathogen that is difficult to treat and eradicate, because it has evolved multiple mechanisms of resistance categorized as intrinsic, acquired, or adaptive [3]. In fact, it has an extraordinary ability to develop resistance to nearly all antimicrobials available either by chromosomal mutations or by acquisition of localized genes in transferable elements [4].

*P. aeruginosa* remains one of the major causes of healthcare-associated infection in Europe, and according to the EARS-Net report 2019, in Spain, 12.5% of invasive isolates of *P. aeruginosa* presented combined resistance to ≥ 3 antimicrobial groups [5]. In the ENVIN-HELICS national registry 2019 (Spanish National ICU-Acquired Infection Surveillance Study), *P. aeruginosa* was the second most frequently isolated microorganism, just behind *Escherichia coli*, as a cause of nosocomial infections in intensive care units (ICUs), and the third most frequent (after *E. coli* and *Staphylococcus aureus*) in community-acquired infections requiring ICU admission [6]. Del Barrio-Tofiño et al. [7] published in 2019 the Spanish nationwide survey on *P. aeruginosa* antimicrobial resistance mechanisms and molecular epidemiology. This study showed that up to 26.2% of the isolates were classified as multidrug-resistant (MDR: non-susceptibility to at least one agent in at least three antibiotic classes), 17.3% as extensively drug-resistant (XDR: non-susceptibility to at least one agent in all, but one or two antibiotic classes), and 0.1% as pandrug resistant (PDR: non-susceptibility to all agents in all antimicrobial categories). ICU isolates were more frequently MDR/XDR than those from other wards. DTR (difficult-to-treat resistance) is a novel classifier of antimicrobial co-resistance that integrates the impact of resistance into antibiotic choices [8]. Recently, the Infectious Diseases Society of America published a guidance for the treatment of DTR-*P. aeruginosa* [9].

The optimization of the use of antimicrobial agents is one of the five strategic objectives included in the global action plan endorsed in 2015 by the World Health Assembly to face antimicrobial resistance [10]. The major indicator of the effect of the antibiotics (pharmacodynamics, PD) is the minimum inhibitory concentration (MIC), but antimicrobial optimization also requires information about the time evolution of the antibiotic concentration in the patients (pharmacokinetics, PK). Pharmacokinetic/pharmacodynamic (PK/PD) analysis has been applied in recent years to optimize therapy, with the aim of maximizing the efficacy and reducing side effects of new and old antibiotics, as well as minimizing the emergence of resistance [11,12]. Currently, it is clearly established that inadequate exposure to antimicrobials can lead to the amplification of resistant subpopulations. The generation of resistant mutants within a bacterial population is an inevitable event, but it is possible to intervene to avoid or reduce the amplification of such subpopulation, and thus to preserve the activity of antimicrobials [13]. Different strategies have been implemented in hospital care to optimize the antimicrobial dosage regimens [14] and, in this context, PK/PD analysis has become an essential tool to be included in antimicrobial stewardship programs [15].

Therefore, based on the information of the large-scale Spanish nationwide survey on *P. aeruginosa* molecular epidemiology and antimicrobial resistance, the objective of this work was to analyze the overall antimicrobial activity of the antipseudomonal antibiotics considering PK/PD criteria. The antimicrobial therapy optimization by PK/PD analysis was applied separately for ICU and medical ward isolates, as well as distinguishing between respiratory and non-respiratory isolates, to support the selection of the appropriate antimicrobial and predicting the dosage regimen with a higher probability of success. A secondary objective was to evaluate the role of PK/PD analysis to prevent or minimize the emergence of resistance to these antimicrobials.

## 2. Materials and Methods

### 2.1. Antimicrobials and Pharmacokinetic Data

Table 1 and Table 2 show the evaluated antimicrobials, their dosage regimens according to the European Committee on Antimicrobial Susceptibility Testing (EUCAST) [16], and the PK parameters in ICU and medical ward patients, respectively. Population PK parameters were obtained from the literature. Prospective studies performed in critically ill and in medical ward patients with infections were selected.

### 2.2. Microbiological Data

Microbiological data (MIC distributions) were extracted from the Spanish nationwide survey study about *P. aeruginosa* isolates collected from 51 participating hospitals, covering all 17 Spanish regions during October 2017 [7], carried out by the GEMARA-SEIMC/REIPI group [46]. The collection included up to 30 consecutive health care-associated non-duplicated (one per patient) *P. aeruginosa* clinical isolates from respiratory, urinary, blood-stream, skin and soft tissue, and osteo-articular, as well as other sample types, and ICU, medical ward, surgical ward, emergency room, and other sources were recorded for each isolate.

Isolates were classified according to the admission service (ICU or medical wards) and according to the sample type (respiratory and non-respiratory). Respiratory samples were collected from tracheal aspirate, bronchial aspiration, sputum, bronchoalveolar lavage, pleural fluid, and bronchial brushing in ICU. In medical wards, the origin of these samples was the same as that from patients from the ICU and nasopharyngeal aspirate and sputum (from cystic fibrosis patients). Based on the location, 91.5% of isolates were collected outside the ICU (only 8.5% of isolates were from ICU). Most samples from ICU patients were from respiratory sources (58.3%); by contrast, most samples collected from medical ward patients were from non-respiratory sources (69.4%).

Susceptibility was recalculated according to the EUCAST breakpoints 2021 [16]. It is important to consider that, in 2019, EUCAST changed definitions of susceptibility testing categories, and intermediate (I-category) was defined as susceptible at increased exposure. Concerning antipseudomonal antimicrobials included in this study, aztreonam, cefepime, ceftazidime, ciprofloxacin, imipenem, and piperacillin/tazobactam are included in this new I-category. The EUCAST breakpoint for imipenem changed from 8 mg/L in 2018 to 4 mg/L in 2021. Table 3 shows the susceptibility rate of *P. aeruginosa* strains classified by admission service and sample location.

### 2.3. PK/PD Analysis and Monte Carlo Simulation

Depending on the activity pattern of the antimicrobial, three different PK/PD indices have been defined as the best descriptors of clinical efficacy [12]: (i) for concentration-dependent activity antimicrobials, the ratio of the total or free-drug maximum concentration (C_max_) to the MIC (C_max_/MIC) or the area under the total or free-drug concentration–time curve, typically over a 24 h period, to the MIC (AUC_24h_/MIC); (ii) for time-dependent patterns, the percentage of time the free drug concentration remains above the MIC throughout the dosage interval (%ƒT_>MIC_); and (iii) AUC_24h_/MIC concentration-dependent with time-dependence antibiotics [11,47].

Table 4 shows the PK/PD indexes and the magnitude of the targets associated with the success of therapy for each antimicrobial. For time-dependent pattern antimicrobials, steady-state concentration (C_ss_) > 4 × MIC was selected as the primary endpoint to evaluate the suitability of the continuous infusion dosage regimens in ICU patients [48].

PK/PD indices and the defined targets for suppression of the emergence of resistance are presented in Table 5.

With the defined PK/PD targets, the probability of target attainment (PTA) and the cumulative fraction of response (CFR) were calculated by Monte Carlo simulation with Oracle^®^ Crystal Ball Fusion Edition v.11.1.2.3.500 software (Oracle Inc., Redwood City, CA, USA) and using 10,000 random iterations of the data. Logarithmic transformation was applied to the mean and the standard deviation of all pharmacokinetic parameters to normalize their distributions, whereas protein binding was included as a fixed value.

#### 2.3.1. Probability of Target Attainment (PTA) Estimation

PTA is the probability that a specific value of the PK/PD index associated with the efficacy of the antibiotic is achieved at a certain MIC [12]. PTA was calculated using the following equations:

- Time-dependent activity antimicrobials;
- IV infusion.

(1) $${\%ƒT}_{>\mathrm{MIC}}=[(t_{2}+t_{i})-t_{1}]\cdot \;\frac{100}{\tau }$$

where %ƒT_>MIC_ is the proportion of time that the free serum concentration remains above the MIC at steady state (%) over a dosage interval, *t_1_* (h) corresponds to the time at which the free serum concentration reached the MIC during the infusion phase; *t_2_* (h) corresponds to the post-infusion time at which the free serum concentration equaled the MIC in the elimination phase; and τ is the dosage interval. The times *t_1_* and *t_2_* were calculated as follows:

(2) $$t_{1}=\frac{\left(\mathrm{MIC}-fC_{\min,\mathrm{ss}}\right)}{(fC_{\max,\mathrm{ss}}-fC_{\min,\mathrm{ss})}}\;t_{inf}$$

(3) $$t_{2}=\mathrm{Ln}\;\left(\frac{fC_{\max,\mathrm{ss}}}{\mathrm{MIC}}\right)\cdot \;\frac{\mathrm{Vd}}{\mathrm{Cl}}\;$$

where Ke is the elimination rate constant.

The minimum and maximum serum concentrations of unbound drug (mg/L) at steady state, ƒC_min,ss_ and ƒC_max,ss_, respectively, were estimated according to the following equations using the total clearance (Cl), volume of distribution (Vd), infusion time (*t_i_*), dosage interval (τ), total dose administered (D), and unbound fraction (ƒ_u_):

(4) $$ƒC_{\min,\mathrm{ss}}=ƒC_{\max,\mathrm{ss}}\cdot {\;e}^{-\frac{\mathrm{Cl}}{\mathrm{Vd}}-\left(\tau -t_{i}\right)}$$

(5) $$ƒ\;C_{\max,\mathrm{ss}}={ƒ}_{u}\cdot \;\frac{D}{\mathrm{Cl}\cdot \;t_{i}}\cdot (1-e^{-\frac{\mathrm{Cl}}{\mathrm{Vd}}\cdot t_{i}})\;\cdot \;\frac{1}{1-{\;e}^{-\frac{\mathrm{Cl}}{\mathrm{Vd}}\cdot Ʈ}}\;$$

- Continuous infusion

(6) $$C_{\mathrm{ss}}=\frac{k_{0}}{\mathrm{Cl}}$$

where C_ss_ is the steady-state concentration, ko is the infusion rate, and Cl is the total clearance.

- Concentration–time-dependent antimicrobials;
- C_max_/MIC: ratio of the maximum drug plasma concentration divided by the MIC.

(7) $$C_{\max}=\frac{\mathrm{ko}\;\cdot \left(1-e^{\left(-\frac{\mathrm{Cl}}{\mathrm{Vd}}\cdot ti\right)}\right)}{\mathrm{Cl}\;\cdot \left(1-e^{\left(-\frac{\mathrm{Cl}}{\mathrm{Vd}}\cdot \tau \right)}\right)}$$

where ko is the infusion rate, Ke is the elimination rate constant, *t_i_* is the infusion time, Cl is the total clearance, and τ is the dosage interval.

- AUC_24h_/MIC: ratio of the area under the antimicrobial concentration–time curve for 24 h divided by the MIC.

(8) $${\mathrm{AUC}}_{24h}=\;\frac{\;D\;}{\mathrm{Cl}}$$

where AUC_24h_ is the area under the serum concentration–time curve over 24 h.

PTA values (%) were calculated for each dosage regimen for both ICU and medical ward dose regimens for an MIC range from 0.0125 to 512 mg/L. Considering that the actual PTA in an individual patient may be significantly different from what would be concluded from a conventional simulation [60], 95% confidence intervals were calculated as the range from the 2.5th to the 97.5th percentile of the set of estimated values.

The dosage regimens were considered successful if PTA was ≥90%, whereas a PTA ≥80%, but <90% was associated with moderate probabilities of success [61].

#### 2.3.2. Calculation of the Cumulative Fraction of Response (CFR)

CFR is the expected probability of success of a dosage regimen against bacteria in the absence of the specific value of MIC, thus the population distribution of MICs is used [12]. It was calculated using Equation (9):

(9) $$\mathrm{CFR}=\sum_{i-1}^{n}\mathrm{PTAi}\;\times \;\mathrm{Fi}$$

where CFR (%) results from the total sum of the products of the PTA at a certain MIC times the frequency (Fi) of isolates of microorganism exhibiting that MIC over the range of susceptible pathogens. The range of MIC concentrations tested for each antimicrobial includes dilutions below the susceptibility breakpoint; therefore, it is adequate to estimate CFR.

The CMI ranges evaluated for each antimicrobial were as follows: amikacin (2–128 mg/L), aztreonam (2–56 mg/L), cefepime and ceftazidime (1–128 mg/L), ceftazidime/avibactam and ceftolozane/tazobactam (0.5–64 mg/L), ciprofloxacin (0.125–32 mg/L), colistin (0.5–16 mg/L), imipenem (0.5–64 mg/L), meropenem (0.5–128 mg/L), piperacillin/tazobactam (4–512 mg/L), and tobramycin (0.25–64 mg/L).

The 95% confidence intervals were calculated as the range from the 2.5th to the 97.5th percentile of the set of estimated values.

A CFR ≥80%, but <90% was associated with moderate probabilities of success, whereas a CFR ≥90% was considered as optimal against that bacterial population [12].

#### 2.3.3. Calculation of the Joint Probability of PK/PD Target Attainment

Joint PTA, calculated for beta-lactam and beta-lactamase inhibitor combinations, are defined as the simultaneous attainment of each individual PTA [23]. It was calculated by determining first if, in each simulated population, the PTA for the beta-lactamase inhibitor is achieved. If this threshold was met, the joint PTA is considered to be the calculated beta-lactam.

## 3. Results

**Figure 1:**
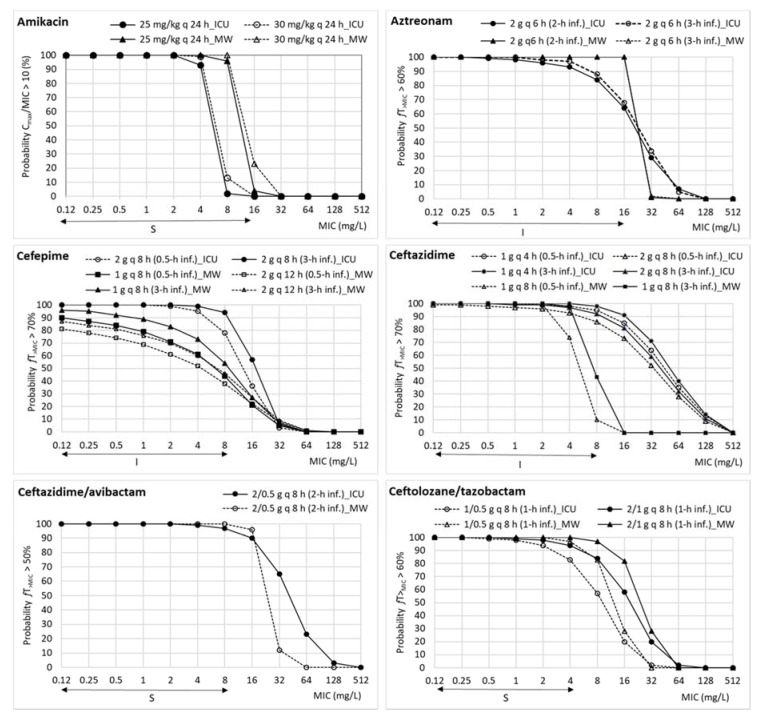
Estimated probability of target attainment (PTA) values for different dosing regimens in both ICU and medical ward patients for amikacin, aztreonam, cefepime, ceftazidime, ceftazidime/avibactam, and ceftolozane/tazobactam. The solid horizontal lines indicate MIC covered by the clinical EUCAST breakpoints (including susceptible and intermediate categories) of *P. aeruginosa* for each antimicrobial. ICU: intensive care unit; MW: medical ward; S: susceptible; I: intermediate category (susceptible at increased exposure).

**Figure 2:**
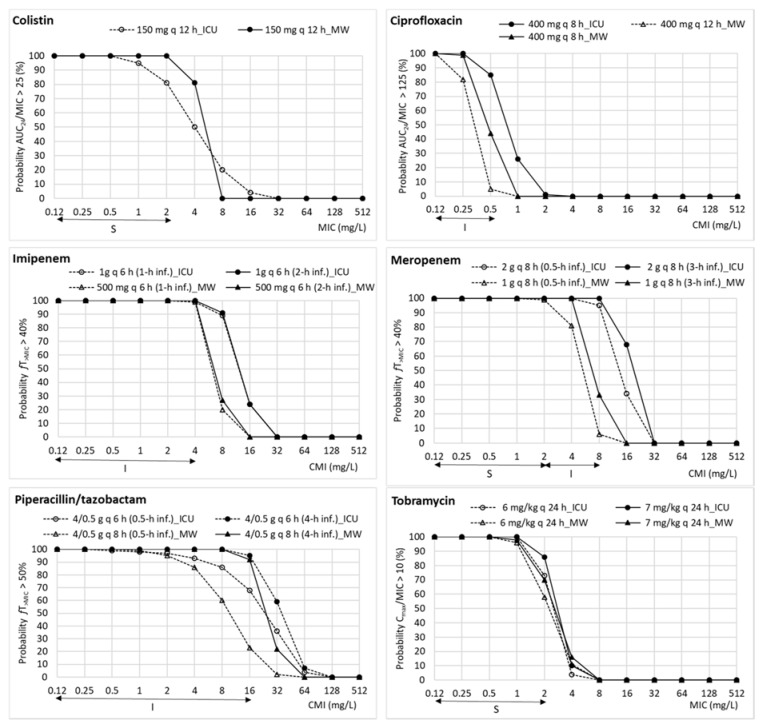
Estimated probability of target attainment (PTA) values for different dosing regimens in both ICU and medical ward patients for colistin, ciprofloxacin, imipenem, meropenem, piperacillin/tazobactam, and tobramycin. The solid horizontal lines indicate MIC covered by the clinical EUCAST breakpoints (including susceptible and intermediate categories) of *P. aeruginosa* for each antimicrobial. ICU: intensive care unit; MW: medical ward; S: susceptible; I: intermediate category (susceptible at increased exposure).

Figure 1 and Figure 2 feature the PTA values calculated by Monte Carlo simulation, considering the EUCAST breakpoints (including susceptible and intermediate categories), at MIC values ranging from 0.125 mg/L to 512 mg/L for each antimicrobial dosage regimen recommended in both ICU and medical wards. PTA values collected in the Supporting Information (Tables S1 and S2) include the 95% confidence interval.

For ICU dosage regimens, PTAs higher than 90% were obtained with ceftazidime/avibactam, cefepime, ceftazidime, ceftolozane/tazobactam imipenem, meropenem, and piperacillin/tazobactam depending on the dose regimen, although, in the case of cefepime, ceftazidime, and piperacillin/tazobactam, only when they are administered as extended infusion. PTA values were under 80% for amikacin and aztreonam, covering MICs up to 4 mg/L and 2 mg/L, respectively, far from the clinical breakpoint (16 mg/L). For medical ward regimen dosages, PTAs higher than 90% were observed for aztreonam, the two new combinations of cephalosporins with beta-lactamase inhibitor, colistin, imipenem, meropenem, and piperacillin/tazobactam, if extended infusion (4 h) is considered. All other antimicrobials showed PTA values under 80% for all dosage regimens evaluated. Tobramycin administered at 7 mg/kg q 24 h showed a PTA value of 72%, although the 95% confidence interval ranged from 69% to 84%, that is, including values corresponding to moderate probabilities of target attainment (>80–90%).

Table 6 features the CFR values obtained, including the 2.5th and 97.5th percentiles, considering the MIC distribution of the isolates classified by admission service and sample location. CFR values estimated from the MIC profile of ICU isolates were below 75% for most antimicrobial regimens, and only ceftazidime/avibactam showed a CFR > 90% (95%), although, with colistin, it was 89%. Only moderate probabilities of success were obtained with ceftolozane/tazobactam (both dosage regimens), ceftazidime (dose 1 g q 4 h, both standard and extended infusion), and meropenem. CFR for amikacin was 71%, despite its high susceptibility values (>90%).

Table 7 shows the probability to reach the suppression of the emergence of antimicrobial resistance, taking into account the MIC distribution of the isolates against the studied antimicrobials. For ICU patients, with the dosage regimens used in clinical practice, no treatment allows to obtain probabilities higher than 90%. Ceftazidime/avibactam reaches a probability of 89% (87–91%) and the highest dose of ceftolozane/tazobactam reaches 86% (84–88%). For medical ward patients, values > 90% were reached only with ceftolozane/tazobactam and ceftazidime/avibactam.

## 4. Discussion

*P. aeruginosa* is among the antimicrobial-resistant gram-negative bacteria challenging current health care. The establishment of programs of antimicrobial activity surveillance integrating local epidemiologic is essential to guide clinicians towards appropriate empiric treatments. The incorporation of PK/PD analysis into these programs affords a valuable complementary tool for a rational antimicrobial and dosage regimen selection. The Spanish nationwide survey on *P. aeruginosa* antimicrobial resistance mechanisms and molecular epidemiology [7] showed that the highest susceptibility rates in both ICU and medical ward isolates were detected for amikacin, ceftazidime/avibactam, ceftolozane/tazobactam, and colistin and, except for these last three antimicrobials, a high prevalence of XDR phenotypes and resistance was documented. In this work, the overall antimicrobial activity of the antipseudomonal antibiotics was assessed by PK/PD analysis to estimate the probability of success of the treatments, incorporating the variability of the pharmacokinetic parameters and the bacterial population. Ceftazidime/avibactam, followed by ceftolozane/tazobactam and colistin, were the most active antimicrobials, with differences depending on the admission service, sample type, and dose regimen. Furthermore, the new combinations of cephalosporins with beta-lactamase inhibitors provided drug exposures associated with resistance suppression at recommended doses.

Standard surveillance indices based on MIC values are insufficient to detect changes in antimicrobial agents´overall activity, as some less obvious variations in MIC distribution may result in treatment efficacy loss. In this regard, different studies on *P. aeruginosa* [40,62,63] highlight that the susceptibility rates and the probability of treatment success estimated by the PK/PD analysis are complementary tools that should be considered together to guide antimicrobial therapy. Our results (Figure 1 and Figure 2) show relevant discrepancies between EUCAST-susceptibility breakpoints and those estimated by PK/PD analysis, defined as the highest MIC value at which a high probability of target attainment is obtained (PTA ≥ 90%). In this sense, the EMA also defends the use of PTA to predict whether a treatment may be useful against a specific microorganism, and underlines its relevance for the treatment of infections caused by multi-resistant bacteria [61]. Besides, in 2019, EUCAST implemented changes to the definitions of susceptibility testing categories to emphasize the relationship between breakpoints and exposure of the organism for the agent at the site of infection, even recommending extended infusions for some time-dependent antimicrobials. As can be observed in Figure 1 and Figure 2 and Supplementary Tables S1 and S2, prolonged infusions of time-dependent antimicrobials enhance PTA against non-susceptible *P. aeruginosa* isolates, thus being a potential therapeutic option for infections due to multidrug-resistant microorganisms. However, in our study, this fact was relevant only for piperacillin/tazobactam. A recent systematic review [64] concluded that, prior to the implementation of prolonged infusion of antipseudomonal beta-lactam regimens, institutions should consider its advantage according to multiple variables including local incidence of *P. aeruginosa* infections, MIC distributions, pharmacokinetic variables, and PTA, as well as implementation challenges.

The Spanish nationwide survey [7] also indicated a complex scenario with major differences in local epidemiology, including carbapenemase production, that need to be acknowledged in order to guide antimicrobial therapy. The estimation of CFRs allows estimating the probability of success for a treatment without knowledge of the susceptibility of the specific isolate responsible for the infection, but taking into account the MIC distribution of a particular institution or hospital wards or regions/countries [11]. Overall, a lack of concordance between susceptibility and CFR values was detected, especially relevant in ICU. With the exception of amikacin, discordances in ICU show higher values of CFR than susceptibility rates. Therefore, taking into account the MIC distribution of UCI isolates in Spain, PK/PD analysis predicts, for the recommended dose regimens, a probability of treatment success higher than that expected if only the susceptibility data are considered. On the contrary, considering the isolates from medical wards, when discrepancies are detected, susceptibility percentages are higher than the CFRs calculated, which may justify treatment failures when dosing selection is based on the susceptibility rate without considering the antibiotic exposure. These results emphasize the importance of taking into account the susceptibility MIC distribution of the isolates of the geographical area or hospital setting and the PK/PD analysis to support empiric therapy.

Monte Carlo simulations were also performed to calculate CFR in ICU for time-dependent antimicrobials administered as continuous infusion at the highest doses, except for ceftazidime/avibactam, with a CFR value of 95% at the recommended dose, and imipenem due to stability concerns. All antimicrobials evaluated, except for ceftolozane/tazobactam (CFR 85%), provided low CFR values (< 60%) at the PK/PD endpoint of C_ss_ > 4 × MIC. The low values could be due to the restrictive target selected; however, this endpoint would allow for maximal bacterial killing and protection against bacterial regrowth considering that critically ill patients are vulnerable to suboptimal dosage and represent a source of selection of resistance to antibiotics [52]. These results agree with those obtained in other studies [65] reporting the target attainment of beta-lactam antibiotics in critically ill patients.

Regarding sample location, recently, Abuhussain et al. [66] conducted a study on the in vitro potency of antipseudomonal beta-lactams against blood and respiratory isolates of *P. aeruginosa* from ICU and non-ICU patients; these authors concluded that the blood sample isolates were more susceptible. In our study, no relevant differences were observed in susceptibility and CFR values between respiratory and non-respiratory isolates, although, for ceftolozane/tazobactam, the susceptibility of respiratory ICU strains was higher than that of non-respiratory ones. In this regard, for ICU isolates, respiratory and non-respiratory, ceftazidime/avibactam was able to attain CFR > 90%, and a high dose of ceftolozane/tazobactam, indicated for hospital-acquired pneumonia including ventilator-associated pneumonia [24], provided CFR > 90% only for respiratory infections.

As a final point, PK/PD analysis based on the MIC distributions of the Spanish national survey was applied to estimate the probability of the suppression of the emergence of resistance to the different antimicrobial dosage regimens. Different works have reported the required antimicrobial PK/PD indices to suppress the emergence of *P. aeruginosa* antibiotic resistance [53,54,55,56,57,58,59], although no standardized methods are currently established to determine the antibiotic exposure for the attainment of resistance suppression. In our study, none of the dosage regimens commonly used in ICU patients were able to attain high probabilities of resistance suppression, although ceftazidime/avibactam and ceftolozane/tazobactam 2/1 g q 8 h provide moderate probabilities (>80–90%). These new antimicrobial combinations were the only ones able to provide concentrations associated with the suppression of resistance at dosage regimens recommended for medical ward (CFR > 90%). Different studies have also concluded that the exposure required for resistance suppression is usually much higher than that to assess the treatment efficacy [11,54]. Consequently, to avoid resistance, the use of alternative dosage strategies should be considered, such as extended or continuous infusions, or the use of some antibiotic combinations for which in vitro studies have demonstrated a clear advantage [47].

With the aim of emphasizing the importance of antimicrobial appropriate use, supporting the development of tools for antibiotic management, and to reduce bacterial resistance, in 2019, the WHO released the Access, Watch, Reserve (AWaRe) classification [67], which categorizes the antibiotics into different stewardship groups. All antipseudomonals evaluated in this study are included in the Watch or Reserve category, except amikacin (Access). Apart from aztreonam, three other antimicrobials are held in reserve, ceftazidime/avibactam, ceftolozane/tazobactam, and colistin, which, in this work, were found to be the three most active against *P. aeruginosa* in terms of susceptibility and CFR, and only the two combinations are able to suppress the emergence of resistance at standard doses.

Finally, this study presents some limitations: (i) PK/PD analysis was carried out using the mean PK parameter and their variability, without considering the possible influence of covariates on the PK behavior of the drugs; (ii) PK information was extracted from studies carried out in critically ill patients and in hospital ward patients and available in the literature; and (iii) many of existing studies determining the exposure required to suppress the emergence of resistance were conducted in vitro, and we have not found the PK/PD indices required to suppress specifically the emergence of resistance of *P. aeruginosa* for all antimicrobials.

## 5. Conclusions

Considering the susceptibility rate and PK/PD criteria, the most active antimicrobial against *P. aeruginosa* was ceftazidime/avibactam, followed by ceftolozane/tazobactam and colistin, all of them categorized as Reserve by the AWaRe WHO classification. Noteworthy discrepancies between EUCAST-susceptibility breakpoints for *P. aeruginosa* and those estimated by PK/PD analysis were observed, as well as a lack of concordance between *P. aeruginosa* isolates susceptibility and CFR values. Our results also highlight the importance of considering the local susceptibility profile, such as the admission service of the patient or the sample location, as well as the PK/PD analysis to support empiric therapy. In this sense, prolonged infusions of time-dependent antimicrobials enhance PTA against non-susceptible *P. aeruginosa* isolate. Furthermore, antimicrobial stewardship programs need to consider not only the efficacy, but also the capacity to suppress resistance emergence to select the proper treatment, in terms of optimal choice of drug and dosage regimen. In this work, based on PK/PD analysis, only standard doses of ceftazidime/avibactam and ceftolozane/tazobactam provided drug concentrations associated with resistance suppression, confirming the need to use different dosage regimens or alternative therapeutic strategies to prevent or minimize resistance emergence.

## Figures and Tables

**Table 1:** Pharmacokinetic parameters for each antimicrobial agent from published studies among critically ill patients (mean ± standard deviation).

Antimicrobial Agent	Dosing Regimen	Infusion Time (h)	Vd (L)	Cl (L/h)	Ke (h^−1^)	Fu	References
Amikacin	25–30 mg/kg q 24 h	0.5	36.27 ± 8.34	5.58 ± 1.56			[17]
Aztreonam	2 g q 6 h	2	27.20 ± 20.80	9.60 ± 5.00		0.72	[18]
		3					
Cefepime	2 g q 8 h	0.5	21.80 ± 5.10	7.62 ± 1.98		0.85	[19,20]
		3					
Ceftazidime	2 g q 8 h	0.5	18.90 ± 9.00		0.27 ± 0.21	0.80	[21]
	1 g q 4 h	3					
Ceftazidime/ avibactam	2/0.5 g q 8 h	2	34.78 ± 10.49	6.14 ± 3.80		0.90	[22,23,24]
			50.81 ± 14.32	11.09 ± 6.78		0.92	
Ceftolozane/ tazobactam	1/0.5 g q 8 h 2/1 g q 8 h	1	20.40 ± 3.70	7.20 ± 3.20		0.79	[25,26]
			32.40 ± 10.00	25.40 ± 9.40		0.70	
Ciprofloxacin	400 mg q 8 h	1		13.60 ± 5.80			[27]
Colistin	150 mg q 12 h	0.5		2.92 ± 2.72			[28]
Imipenem	1 g q 6 h	1	28.70 ± 9.70	11.40 ± 3.53		0.80	[29]
		2					
Meropenem	2 g q 8 h	0.5	22.70 ± 3.70	13.60 ± 2.08		0.98	[30]
		3					
Piperacillin/ tazobactam	4/0.5 g q 6 h	0.5	19.40 ± 7.76	13.80 ± 4.77		0.75	[31]
		4					
Tobramycin	6–7 mg/kg q 24 h	0.5	17.50 ± 5.25		0.25 ± 0.01		[32]

Vd: volume of distribution; Cl: total clearance; Ke: elimination rate constant; Fu: unbound fraction.

**Table 2:** Dosing regimen and pharmacokinetic parameters for each antimicrobial agent from published studies in non-critically ill patients (mean ± standard deviation).

Antimicrobial Agent	Dosing Regimen	Infusion Time (h)	Vd (L)	Cl (L/h)	AUC (mg/L · h)	Fu	References
Amikacin	25–30 mg/kg q 24 h	0.5	15.80 ± 3.50	5.87 ± 0.98			[33]
Aztreonam	2 g q 6 h	2	0.14 ± 0.04 (L/kg)	4.41 ± 0.63		0.40	[34]
		3					
Cefepime	1 g q 8 h	0.5	0.28 ± 0.25 (L/kg)	7.00 ± 4.30		0.80	[35]
	2 g q 12 h	3					
Ceftazidime	1 g q 8 h	0.5	15.75 ± 1.50	6.96 ± 1.08		0.90	[36]
		3					
Ceftazidime/ avibactam	2/0.5 g q 8 h	2	18.70 ± 1.65	7.53 ± 1.28		0.90	[24,37]
			25.30 ± 4.43	12.30 ± 1.96		0.92	
Ceftolozane/ tazobactam	1/0.5 g q 8 h 2/1 g q 8 h	1	13.50 ± 2,83	4.76 ± 1.13		0.79	[38,39,40]
			18.20 ± 4.55	20.51 ± 4.40		0.70	
Ciprofloxacin	400 mg q 12 h	1			20.80 ± 5.70		[41]
Colistin	150 mg q 12 h	0.5		2.92 ± 0.10			[42]
Imipenem	500 mg q 6 h	1	16.50 ± 3.75	10.50 ± 1.38		0.90	[43]
		2					
Meropenem	1 g q 8 h	0.5	20.25 ± 3.00	14.40 ± 1.80		0.92	[43]
		3					
Piperacillin/ tazobactam	4/0.5 g q 8 h	0.5	11.25 ± 1.50	10.22 ± 2.12		0.70	[43]
		4					
Tobramycin	6–7 mg/kg q 24 h	0.5	20.50 ± 11.40	5.19 ± 0.91			[44,45]

Vd: volume of distribution; Cl: total clearance; AUC: area under the curve; Fu: unbound fraction.

**Table 3:** Percentage of *P. aeruginosa* susceptible strains in 2017, classified by admission service and sample location, according to the Spanish nationwide survey [7] and applying EUCAST clinical breakpoints [16].

	Susceptibility (%)					
	ICU			Medical Ward Patients		
Antimicrobial Agent and Dosing Regimen	Total	Respiratory	Non-Respiratory	Total	Respiratory	Non-Respiratory
Amikacin	91 **	93 **	90 **	92 **	96 **	97 **
Aztreonam	70	64	77	*87*	*87*	*87*
Cefepime	67	69	65	*80*	72	82
Ceftazidime	64	67	60	*81*	79	83
Ceftazidime/avibactam	*85*	*87*	*83*	95 **	96 **	95 **
Ceftolozane/ tazobactam	*81*	*87*	73	96 **	95 **	96 **
Ciprofloxacin	52	46	60	62	55	65
Colistin	95 **	96 **	94 **	95 **	96 **	94 **
Imipenem	55	57	52	75	72	75
Meropenem	71	73	69	*80*	*87*	*87*
Piperacillin/ tazobactam	57	57	58	75	73	76
Tobramycin	74	75	73	*84*	*84*	*85*

** susceptibility ≥90%; *underlined and in italics*, susceptibility ≥80% and <90%.

**Table 4:** Pharmacokinetic/pharmacodynamics (PK/PD) index and target magnitude for each antimicrobial agent.

Antimicrobial Agent	PK/PD Target	References
Amikacin	C_max_/MIC > 10	[49]
Aztreonam	%ƒT_>MIC_ > 60	[18]
Cefepime	%ƒT_>MIC_ > 70	[50]
Ceftazidime	%ƒT_>MIC_ > 70	[12]
Ceftazidime/ avibactam	%ƒT_>MIC_ > 50% %ƒT > 1 mg/L > 50%	[23]
Ceftolozane/ tazobactam	%ƒT_>MIC_ > 60% %ƒT > 1 mg/L > 20%	[25,26]
Ciprofloxacin	*f*AUC_24h_/MIC > 125	[51]
Colistin	*f*AUC_24h_/MIC > 25–35	[52]
Imipenem	%ƒT_>MIC_ > 40	[12]
Meropenem	%ƒT_>MIC_ > 40	[51]
Piperacillin/tazobactam	%ƒT_>MIC_ > 50	[41]
Tobramycin	C_max_/MIC > 10	[36]
Time-dependent antimicrobials Continuous infusion	Css > 4 × MIC	[48]

%ƒT_>MIC_: Percentage of time that the antimicrobial free serum concentration remained above the MIC; %*f*T > 1 mg/L: cumulative percentage over a 24 h period that the free drug concentration exceeded a 1 mg/L threshold concentration; ƒAUC_24h_: area under the free drug concentration–time curve over a 24 h period; C_max_: maximum drug plasma concentration; MIC: minimum inhibitory concentration; C_ss_: steady-state concentration.

**Table 5:** The pharmacokinetic/pharmacodynamic (PK/PD) indices reported to suppress the emergence of antibiotic resistance for *P. aeruginosa*.

Antimicrobial	PK/PD Index	PK/PD Index Magnitude		References
		Total Drug	Free Drug	
Cefepime	C_min_/MIC		≥ 3.8	[53]
Ceftazidime	%ƒT_>MIC_		≥ 100	[54]
Ceftazidime/avibactam	%ƒT_>MIC_		≥ 87	[55]
Ceftolozane/tazobactam	%ƒT_>MIC_	≥ 80		[56]
Piperacillin/tazobactam	C_min_/MIC		≥ 5	[57]
Meropenem	C_min_/MIC		≥ 3.8	[53]
Imipenem	AUC_24_/MIC	= 140		[58]
Ciprofloxacin	*f*AUC_24_/MIC	≥ 385		[59]

C_min_: minimum concentration; MIC: minimum inhibitory concentration; %ƒT_>MIC_: percentage of time that the antimicrobial-free serum concentration remains above the MIC; AUC_24h_: area under the concentration–time curve from 0 h to 24 h.

**Table 6:** Cumulative fraction of response (CFR) calculated for all dosing regimens classified by admission service and sample location. Numbers in parentheses indicate the 2.5th and 97.5th percentiles.

Antimicrobial Agent and Dosing Regimen	CFR (%)					
	ICU			Medical Ward Patients		
Amikacin	Total	Respiratory	Non-Respiratory	Total	Respiratory	Non-Respiratory
25 mg/kg q 24 h	71 (68–74)	77 (75–79)	63 (60–66)	92 (91–94) **	88 (86–90) *	94 (93–96) **
30 mg/kg q 24 h	72 (69–75)	81 (78–84)	67 (64–70)	92 (91–94) **	90 (88–92) **	95 (94–97) **
Aztreonam						
2 g q 6 h (2 h inf.)	65 (62–69)	62 (59–65)	70 (67–73)	86 (83–87) *	85 (83–87) *	87 (84–89) *
2 g q 6 h (3 h inf.)	69 (66–71)	66 (64–70)	71 (68–74)	86 (84–88) *	85 (83–87) *	87 (85–89) *
Cefepime						
1 g q 8 h (0.5 h inf.)				57 (54–60)	51 (48–54)	53 (50–57)
2 g q 12 h (0.5 h inf.)				48 (45–51)	45 (42–48)	46 (43–49)
2 g q 8 h (0.5 h inf.)	68 (71–61)	67 (64–69)	65 (62–68)			
1 g q 8 h (3 h inf.)				63 (59–66)	59 (56–62)	65 (62–68)
2 g q 12 h (3 h inf.)				53 (49–55)	53 (51–55)	55 (53–59)
2 g q 8 h (3 h inf.)	77(75–80)	76 (73–78)	73 (70–75)			
Ceftazidime						
1 g q 4 h (0.5 h inf.)	80 (78–83) *	83(81–86) *	78 (75–81)			
1 g q 8 h (0.5 h inf.)				68 (65–70)	63 (60–66)	64 (60–66)
2 g q 8 h (0.5 h inf.)	76 (74–79)	76 (73–79)	72 (69–75)			
1 g q 4 h (3 h inf.)	85 (82–87) *	85 (83–87)	81 (78–83) *			
1 g q 8 h (3 h inf.)				73 (71–76)	75 (72–77)	74 (72–77)
2 g q 8 h (3 h inf.)	79 (76–82)	81 (79–83) *	77 (74–80)			
Ceftazidime/avibactam						
2/0.5 g q 8 h (2 h inf.)	95 (94–97) **	98 (97–99) **	93 (92–95) **	97 (96–98) **	98 (97–98) **	97 (96–99) **
Ceftolozane/tazobactam						
1/0.5 g q 8 h (1 h inf.)	84 (81–86) *	85 (82–87) *	81 (78–83) *	95 (94–96) **	92 (91–94) **	96 (95–98) **
2/1 g q 8 h (1 h inf.)	86 (84–88) *	92 (91–94) **	83 (81–86) *	96 (95–97) **	95 (94–96) **	97 (95–98) **
Ciprofloxacin						
400 mg q 12 h				53 (50–57)	43 (40–47)	58 (55–61)
400 mg q 8 h	54 (51–57)	48 (45–50)	59 (56–63)			
Colistin						
150 mg q 12 h	89 (86–90) *	88 (86–90) *	88 (87–90) *	95 (94–96) **	95 (94–97) **	94 (93–95) **
Imipenem						
500 mg q 6 h (1 h inf.)				75 (72–78)	75 (73–77)	78 (76–80)
1 g q 6 h (1 h inf.)	77 (74–79)	83 (81–85) *	77 (75–80)			
500 mg q 6 h (2 h inf.)				77 (75–80)	75 (73–78)	78 (75–80)
1 g q 6 h (2 h inf.)	81 (79–84) *	83 (81–85) *	76 (73–79)			
Meropenem						
1 g q 8 h (0.5 h inf.)				79 (77–82)	80 (77–82) *	80 (78–84) *
2 g q 8 h (0.5 h inf.)	77 (74–80)	79 (77–82)	73 (71–76)			
1 g q 8 h (3 h inf.)				84 (81–86) *	82 (80–84) *	83 (81–86) *
2 g q 8 h (3 h inf.)	82 (79–84) *	86 (84–88) *	77 (75–80)			
Piperacillin/tazobactam						
4/0.5 g q 8 h (0.5 h inf.)				51 (48–54)	50 (46–54)	50 (47–53)
4/0.5 g q 6 h (0.5 h inf.)	53 (49–56)	52 (49–56)	55 (52–59)			
4/0.5 g q 8 h (4 h inf.)				79 (76–82)	75 (73–78)	76 (74–79)
4/0.5 g q 6 h (4 h inf.)	64 (61–67)	67 (64–70)	68 (64–70)			
Tobramycin						
6 mg/kg q 24 h	72 (69–75)	72 (69–75)	71 (68–75)	81 (79–84) *	80 (78–83) *	81 (79–83) *
7 mg/kg q 24 h	70 (67–73)	71 (69–74)	72 (69–75)	82 (79–84) *	82 (79–84) *	83 (81–85) *

** susceptibility ≥90%; * susceptibility ≥80% and <90%; inf: infusion time.

**Table 7:** Cumulative fraction of response (CFR) calculated for all dosing regimens considering PK/PD indices to suppress the emergence of resistance. Numbers in parentheses indicate the 2.5th and 97.5th percentiles.

	CFR (%)	
Antimicrobial Agent and Dosing Regimen	ICU	Medical Ward Patients
Cefepime		
1 g q 8 h (0.5 h inf.)		20 (18–23)
2 g q 12 h (0.5 h inf.)		14 (12–16)
2 g q 8 h (0.5 h inf.)	10 (9–12)	
1 g q 8 h (3 h inf.)		19 (17–22)
2 g q 12 h (3 h inf.)		16 (14–19)
2 g q 8 h (3 h inf.)	19 (17–21)	
Ceftazidime		
1 g q 4 h (0.5 h inf.)	78 (76–81)	
1 g q 8 h (0.5 h inf.)		26 (23–29)
2 g q 8 h (0.5 h inf.)	65 (62–68)	
1 g q 4 h (3 h inf.)	81 (79–84) *	
1 g q 8 h (3 h inf.)		51 (48–54)
2 g q 8 h (3 h inf.)	69 (66–72)	
Ceftazidime/avibactam		
2/0.5 g q 8 h (2 h inf.)	89 (87–91) *	91 (90–93) **
Ceftolozane/tazobactam		
1/0.5 g q 8 h (1 h inf.)	77 (75–80)	96 (95–97) **
2/1 g q 8 h (1 h inf.)	86 (84–88) *	97 (97–98) **
Ciprofloxacin		
400 mg q 12 h		0 (0–0)
400 mg q 8 h	36 (33–39)	
Imipenem		
500 mg q 6 h		0 (0–0)
1 g q 6 h	42 (39–45)	
Meropenem		
1 g q 8 h (0.5 h inf.)		0 (0–0)
2 g q 8 h (0.5 h inf.)	5 (4–7)	
1 g q 8 h (3 h inf.)		1 (0–2)
2 g q 8 h (3 h inf.)	15 (13–18)	
Piperacillin/tazobactam		
4/0.5 g q 8 h (0.5 h inf.)		0 (0–0)
4/0.5 g q 6 h (0.5 h inf.)	2 (1–3)	
4/0.5 g q 8 h (4 h inf.)		0 (0–0)
4/0.5 g q 6 h (4 h inf.)	8 (7–10)	

** CFR ≥ 90%; * CFR ≥ 80% and < 90%; inf: infusion time.

## Data Availability

All data are available in the main text.

## Supplementary Materials

The following are available online at https://www.mdpi.com/article/10.3390/pharmaceutics13111899/s1, Table S1: Probability target attainment (PTA) (%) at each value of minimum inhibitory concentration (MIC) in ICU patients. Numbers in parenthesis indicate the 2.5th and 97.5th percentiles. The solid vertical lines indicate the intercept with the EUCAST clinical breakpoints of *P aeruginosa*. Grey shading indicates PTA > 90%. Table S2: Probability target attainment (PTA) (%) at each value of minimum inhibitory concentration (MIC) in medical ward patients. Numbers in parenthesis indicate the 2.5th and 97.5th percentiles. The solid vertical lines indicate the intercept with the EUCAST clinical breakpoints of *P aeruginosa*. Grey shading indicates PTA > 90%.
