# Supplementary material for: Pseudomonas aeruginosa Susceptibility in Spain: Antimicrobial Activity and Resistance Suppression Evaluation by PK/PD Analysis
Source: Pharmaceutics. 2021 Nov 8;13(11):1899. doi: 10.3390/pharmaceutics13111899 (PMC8620410; doi:10.3390/pharmaceutics13111899)
Supplement: Supplementary file 1 [file pharmaceutics-13-01899-s001.zip › pharmaceutics-1418153-supplementary.pdf]

pharmaceutics

MDPI

Supplementary Materials: *Pseudomonas Aeruginosa* Susceptibility in Spain: Antimicrobial Activity and Resistance Suppression Evaluation by PK/PD Analysis

Ana Valero, Alicia Rodríguez-Gascón, Arantxa Isla, Helena Barrasa, Ester del Barrio-Tofiño, Antonio Oliver, Andrés Canut and María Ángeles Solinís

Table S1. Probability target attainment (PTA) (%) at each value of minimum inhibitory concentration (MIC) in ICU patients. Numbers in parenthesis indicate the 2.5th and 97.5th percentiles. The solid vertical lines indicate the intercept with the EUCAST clinical breakpoints of *P aeruginosa*. Grey shading indicates PTA > 90%.

	MIC (mg/L)											
**Antimicrobial Agent and Dosing Regimen**	**0.06**	**0.12**	**0.25**	**0.5**	**1**	**2**	**4**	**8**	**16**	**32**	**64**	**128**
Amikacin												
25 mg/kg q 24 h	100	100	100	100	100	100	93 (91—4)	2 (1–3)	0	0	0	0
30 mg/kg q 24 h	100	100	100	100	100	100	99 (99–100)	11 (9–14)	0	0	0	0
Aztreonam												
2 g q 6 h (2 h inf.)	100	100	100 (99–100)	98 (98–99)	98 (97–99)	96 (94–97)	93 (91–94)	84 (82–87)	64 (61–67)	29 (27–32)	7 (5–8)	0
2 g q 6 h (3-h inf.)	100	100	100	100	100	98 (98–99)	97 (96–98)	88 (85–90)	68 (65–71)	34 (31–37)	5 (4–6)	0
Cefepime												
2 g q 8 h (0.5-h inf.)	100	100	100	100	100	99 (98–99)	95 (94–96)	76 (74–79)	34 (32–37)	3 (2–4)	0	0
2 g q 8 h (3-h inf.)	100	100	100	100	100	100	100	93 (92–95)	57 (54–60)	6 (4–7)	0	0
Ceftazidime												

1 g q 4 h (0.5 h inf.)	100	100	100	100	100	100 (99–100)	98 (97–99)	96 (94–97)	85 (83–88)	64 (61–67)	35 (32–38)	12 (11–14)
2g q8h (0.5-h inf.)	100	100 (99–100)	99 (99–100)	99 (99–100)	98 (98–99)	96 (97–98)	92 (90–93)	86 (84–88)	75 (72–77)	53 (50–57)	28 (25–30)	10 (8–12)
	**MIC (mg/L)**											
**Antimicrobial agent and dosing regimen**	**0.06**	**0.12**	**0.25**	**0.5**	**1**	**2**	**4**	**8**	**16**	**32**	**64**	**128**
Ceftazidime												
1 g q 4 h (3-h inf.)	100	100	100	100	100	100	100	98 (97–99)	92 (90–94)	72 (69–75)	38 (35–41)	15 (13–18)
2 g q 8 h (3-h inf.)	100	100	100	100	99 (99–100)	99 (98–100)	96 (95–97)	92 (90–93)	81 (79–84)	60 (57–63)	34 (31–37)	10 (8–11)
Ceftazidime/avibactam												
2/0.5 g q 8 h (2 h inf.)	100	100	100	100	100	100	100 (99–100)	97 (97–98)	91 (89–92)	63 (60–66)	22 (19–24)	3 (2–4)
Ceftolozane/tazobactam												
1/0.5 g q 8 h (1-h inf.)	100	100	100	100 (99–100)	98 (97–99)	94 (92–95)	84 (82–87)	55 (52–58)	21 (18–23)	1 (1–2)	0	0
2/1 g q 8 h (1-h inf.)	100	100	100	100	99 (99–100)	97 (96–98)	95 (94–96)	85 (83–87)	57 (54–60)	20 (18–22)	1 (1–2)	0
Ciprofloxacin												
400 mg q 8 h	100	100	100	85 (83–88)	27 (24–29)	1 1–2	0	0	0	0	0	0
Colistin												
150 mg q 12 h	100	100	100	100 (99–100)	95 (94–98)	81 (77–85)	50 (45–56)	20 (15–23)	4 (2–6)	0	0	0
	**MIC (mg/L)**											

**Antimicrobial agent and dosing regimen**	**0.06**	**0.12**	**0.25**	**0.5**	**1**	**2**	**4**	**8**	**16**	**32**	**64**	**128**
Imipenem												
1 g q 6 h (1-h inf.)	100	100	100	100	100	100	100 (99–100)	89 (87–91)	24 (22–27)	0	0	0
1 g q6h (2 h inf.)	100	100	100	100	100	100	100	91 (89–92)	26 (24–29)	0	0	0
Meropenem												
2 g q 8 h (0.5 h inf.)	100	100	100	100	100	100	100	95 (94–97)	34 (31–37)	0	0	0
2 g q 8 h (3-h inf.)	100	100	100	100	100	100	100	100	68 (65–71)	0	0	0
Piperacillin/tazobactam												
4/0.5 g q6h (0.5-h inf.)	100	100	100	99 (98–99)	98 (97–99)	97 (96–98)	93 (91–94)	84 (82–87)	69 (66–72)	35 (33–38)	5 (4–6)	0
4/0.5 g q 6 h (4 h inf.)	100	100	100	100	100	100	100	100	94 (93–96)	58 (55–61)	6 (5–7)	0
Tobramycin												
6 mg/kg q 24 h	100	100	100	100	100 (99–100)	71 (66–77)	4 (2–6)	0	0	0	0	0
7 mg/kg q 24 h	100	100	100	100	100	86 (82–90)	10 (8–14)	0	0	0	0	0

Table S2. Probability target attainment (PTA) (%) at each value of minimum inhibitory concentration (MIC) in medical ward patients. Numbers in parenthesis indicate the 2.5th and 97.5th percentiles. The solid vertical lines indicate the intercept with the EUCAST clinical breakpoints of *P aeruginosa*. Grey shading indicates PTA > 90%.

	**MIC (mg/L)**											
**Antimicrobial Agent and Dosing Regimen**	**0.06**	**0.12**	**0.25**	**0.5**	**1**	**2**	**4**	**8**	**16**	**32**	**64**	**128**
Amikacin												
25 mg/kg q 24 h	100	100	100	100	100	100	100	95 (94–97)	3 (2–4)	0	0	0
30 mg/kg q 24 h	100	100	100	100	100	100	100	100	18 (16–21)	0	0	0
Aztreonam												
2 g q 6 h (2 h inf.)	100	100	100	100	100	100	100	100 (99–100)	91 (90–92)	4 (2–6)	0	0
2 g q 6 h (3-h inf.)	100	100	100	100	100	100	100	100	96 (94–98)	6 (4–8)	0	0
Cefepime												
1 g q 8 h (0.5-h inf.)	92 (90–94)	91 (89–92)	86 (84–88)	84 (81–86)	81 (79–83)	71 (69–74)	60 (57–63)	42 (39–45)	23 (21–25)	4 (3–6)	1 (0–1)	0
2 g q 12 h (0.5-h inf.)	86 (84–88)	83 (81–85)	77 (75–80)	75 (72–78)	68 (65–71)	63 (60–65)	53 (50–56)	39 (35–42)	23 (20–26)	8 (7–10)	1 (1–2)	0
1 g q 8 h (3-h inf.)	97 (96–98)	96 (95–97)	94 (93–96)	92 (89–93)	90 (88–92)	84 (82–86)	71 (69–74)	54 (49–57)	29 (26–32)	7 (6–9)	0 (0–1)	0
2 g q 12 h (3 h inf.)	9 (87–91)	87 (86–90)	82 (80–85)	82 (80–85)	76 (74–79)	70 (67–73)	60 (56–63)	47 (44–50)	27 (24–29)	9 (7–11)	1 (1–2)	0

	**MIC (mg/L)**											
**Antimicrobial Agent and Dosing Regimen**	**0.06**	**0.12**	**0.25**	**0.5**	**1**	**2**	**4**	**8**	**16**	**32**	**64**	**128**

Ceftazidime												
1 g q 8 h (0.5-h inf.)	100	100	100	100	100	98 (98–99)	76 (73–78)	9 (7–11)	0	0	0	0
1 g q 8 h (3-h inf.)	100	100	100	100	100	100	99 (97–99)	44 (42–47)	0	0	0	0
Ceftazidime/avibactam												
2/0.5 g q 8 h (2 h inf.)	100	100	100	100	100	100	100	100	96 (95–97)	12 (10–15)	0	0
Ceftolozane/tazobactam												
1/0.5 g q 8 h (1-h inf.)	100	100	100	100	100	100	98 (98–99)	85 (83–88)	31 (28–34)	1 (0–1)	0	0
2/1 g q8h (1 h inf.)	100	100	100	100	100	100	100	99 (98–99)	85 (83–87)	32 (30–35)	0	0
Ciprofloxacin												
400 mg q 12 h	100	100	81 (79–84)	4 (2–5)	0	0	0	0	0	0	0	0
400 mg q 8 h	100	100	99 (99–100)	42 (39–45)	0	0	0	0	0	0	0	0
Colistin												
150 mg q 12 h	100	100	100	100	100	100	81 (78–83)	0	0	0	0	0

	MIC (mg/L)											
**Antimicrobial Agent and Dosing Regimen**	**0.06**	**0.12**	**0.25**	**0.5**	**1**	**2**	**4**	**8**	**16**	**32**	**64**	**128**
Imipenem												
500 mg q 6 h (1-h inf.)	100	100	100	100	100	100	99 99–100	19 17–22	0	0	0	0

500 mg q 6 h (2-h inf.)	100	100	100	100	100	100	100	26 (22–29)	0	0	0	0
Meropenem												
1g q 8 h (0.5-h inf.)	100	100	100	100	100	99 (99–100)	80 (78–82)	6 (5–8)	0	0	0	0
1g q 8 h (3-h inf.)	100	100	100	100	100	100	100	33 (31–37)	0	0	0	0
Piperacillin/tazobactam												
4/0.5 g q 8 h (0.5 h inf.)	100	100	100	100	99 (98–100)	95 (94–97)	84 (82–87)	57 (54–60)	20 (18–23)	2 (1–3)	0	0
4/0.5 g q 8 h (4 h inf.)	100	100	100	100	100	100	100	100	92 (90–93)	21 (19–24)	0	0
Tobramycin												
6 mg/kg q 24 h	100	100	100	100	95 (93–96)	56 (53–58)	11 (9–13)	0	0	0	0	0
7 mg/kg q 24 h	100	100	100	100	98 (98–99)	72 (69–84)	17 (14–20)	1 (0–1)	0	0	0	0
